# Phytochemical Nanoparticles for the Treatment of Neurological Disorders

**DOI:** 10.1002/pca.70020

**Published:** 2025-07-29

**Authors:** Touraj Ehtezazi, Satyajit D. Sarker

**Affiliations:** ^1^ Centre for Natural Product Discovery, School of Pharmacy and Biomolecular Sciences Liverpool John Moores University Liverpool UK

**Keywords:** Alzheimer's disease, blood–brain barrier, nanoparticles, neurological disorders, phytochemical

## Abstract

Neurological disorders impose a significant burden on the healthcare systems. The latest published data by WHO indicated that stroke was the second leading cause of death globally in 2020, with Alzheimer's disease (ad) and other dementias in the seventh position. The treatment of neurological disorders is challenging because of the complex nature of the disease, as well as limited accessibility to this target organ due to several biological barriers. There is a wide range of treatment options for neurological disorders. Small drug molecules, antibodies, and stem cells have been employed for the treatment of neurodegenerative diseases such as AD, but currently, there is no effective treatment for AD. As conventional drugs have not been successful in achieving therapeutic outcomes, natural products such as curcumin, stemming from traditional medicines, have been tested for the treatment of neurodegenerative diseases such as AD. However, this compound has not shown significant therapeutic effects for the treatment of brain diseases, mainly due to rapid clearance from the body. Therefore, phytochemical nanoparticles have been developed. In this review article, the rationale has been provided for the use of nanoparticles for the treatment of neurodegenerative diseases with emphasis on phytochemical nanoparticles.

## Abstract 2

Neurological disorders significantly burden healthcare systems, with stroke being the second leading cause of death globally in 2020 and Alzheimer's disease ranking seventh. Treatment is challenging due to disease complexity and biological barriers limiting brain access. Current therapies, including small molecules, antibodies, and stem cells, have proven ineffective for conditions like Alzheimer's. Natural products like curcumin from traditional medicine show promise but suffer from rapid body clearance. Consequently, researchers are developing phytochemical nanoparticles as a potential solution for treating neurodegenerative diseases more effectively.

**Abbreviations**
ad
: Alzheimer's disease
cRGD: cyclic RGD peptide
DOX: doxorubicin
GBM: glioblastoma
GMO: glycerylmonooleate
HD: Huntington's disease
I/R: ischemic reperfusion
IH: intracerebral hemorrhage
IN: intranasal delivery
LN: lipid nanoparticles
MCAO: middle cerebral artery occlusion
MS: multiple sclerosis
MWM: Morris water maze
ND: not determined
NLC: nanostructured lipid carriers
NI: neuroinflammation
PAMAM: poly (amidoamine)
PD: Parkinson's disease
PS‐(PCL): phosphatidylserine‐poly‐ε‐caprolactone
RBC: red blood cell
SAH: subarachnoid hemorrhage
TBI: traumatic brain injury
TG: TGNYKALHPHNG

## 1 Introduction

Neurological disorders are categorized into communicable and noncommunicable disorders. Tetanus, meningitis, and encephalitis are examples of communicable disorders. On the other hand, epilepsy, Alzheimer's disease (ad), Parkinson's disease (PD), multiple sclerosis (MS), stroke, and traumatic brain injury (TBI) are examples of noncommunicable neurological disorders. Recognition of noncommunicable disorders such as epilepsy goes back to 1808 [1]. Neurodegenerative diseases are part of both communicable and noncommunicable neurological disorders with progressive neural function loss, including cognitive and motor functions, leading to a patient's death. The causes of neurological disorders vary widely. Symptoms can range from mild to severe and may include memory loss, tremors, paralysis, seizures, and changes in mood or personality. For example, stroke is caused by the focal injury of the central nervous system by a vascular cause, such as blockage of the vessel by a blood clot (ischemic stroke) or rupture of blood vessels (hemorrhagic stroke). The lack of blood flow causes cerebral tissue necrosis and focal neuronal deficits. In addition, other pathophysiological effects occur, including inflammation [2], increased release of proinflammatory cytokines (TNFα and IL‐6) [3], complement activation [4], and impairment of the blood–brain barrier (BBB) [5], which makes stroke the leading cause of mortality and disability worldwide [6]. Increasing age is the most important risk factor for certain noncommunicable neurological disorders such as stroke [7], PD, and ad [8]. Clinicians prepare a diagnosis based on the patient's medical history and a physical exam, together with other appropriate tests. For example, for ad, diagnosis often requires a combination of neurological examinations (cognitive function tests), imaging techniques like magnetic resonance imaging (MRI) or computed tomography (CT) scans [9], and laboratory tests for measuring biomarkers such as total tau in cerebrospinal fluid [10].

Neurological disorders impose a significant healthcare burden. The latest published data by WHO indicated that stroke was the second leading cause of death globally in 2020, with Alzheimer's disease and other dementia in the seventh position (https://www.who.int/news‐room/fact‐sheets/detail/the‐top‐10‐causes‐of‐death). The impacts of neurological disorders are expressed by disability‐adjusted life years (DALYs), which is the sum of the years that a patient lives with disability and the years that are lost before the life expectancy (due to premature mortality). An estimated 3.40 billion individuals had a condition affecting the nervous system in 2021, corresponding to 43.1% of the world population [11]. These conditions caused 11.1 million deaths [11]. The number of all neurological conditions was 3.4 million in 2021, which was a 58.8% increase from 1990, perhaps due to increased life expectancy. DALY counts increased by 18.2% from 375 million DALYs in 1990 to 443 million DALYs in 2021 for total nervous system health loss [11]. The leading cause of disability changed by age; for example, stroke was the second cause of disability for the 75 years and older age group (with 13% of 329 million total DALYs count), whereas this was the ninth for the 25‐ to 49‐year age group (with 3.2% of 616 million DALYs count) in 2019 [12]. Stroke was the first, and TBI was the 14th noncommunicable neurological disorder in the ranking of age‐standardized DALY rates for all conditions with neurological health loss by GBD region in 2021 [11].

In the majority of neurodegenerative disorders, microglia (major brain‐resident immune cells) are activated, which may lead to the release of proinflammatory cytokines [13, 14]. This promotes infiltration of peripheral immune cells into the central nervous system (CNS) [15, 16], which is collectively known as neuroinflammation. Dysregulated neuronal autophagy and impaired remyelination are examples of neuroinflammation's pathological effects [17, 18]. Although there are compensation mechanisms to repair damaged neurons, these are not sufficient to prevent the progression of the damage. For example, there are axon damages early in MS, and these are compensated by mechanisms such as remyelination. However, lesions in the gray and white matter gradually expand and become prominent. Continuous and low‐level inflammation and loss of compensatory mechanisms result in segmental and global atrophy [19]. The microglia are rapidly activated following TBI and mobilize to the damaged area to clear debris [20]. As there could be potential microbial contamination, microglia release proinflammatory cytokines to induce a cascade of inflammatory response, including infiltration of peripheral immune cells [21]. This is to ensure that the brain environment is suitable for the normal functioning of neurons. However, an uncontrolled and even excessive activation becomes detrimental and promotes neurodegeneration. The dysregulated microglia activation can be maintained up to 17 years in TBI patients [22]. As the BBB is also damaged in TBI, and natural compounds such as phillyrin, from *Forsythia suspensa* (Thunb.) Vahl, also known as Lianqiao in Chinese traditional medicine, could repair the BBB after TBI [23]. Delayed repair of the BBB becomes a contributing factor to neural apoptosis [24]. Recent studies demonstrated that a natural product (ACT001, also known as dimethylamino‐micheliolide, a certified orphan drug) reduced microglia activation in vitro by lipopolysaccharides (LPSs) through suppression of the NFκB/NLRP3 neuroinflammatory pathway by inhibiting the phosphorylation of AKT [14]. These results explained the alleviation of motor function deficits in mice after TBI by administering ACT001 [14].

Phytochemical compounds have long been utilized in traditional medicine for treating neurodegenerative disorders (examples provided in the above), offering a promising approach with an established safety profile. The historical use of these compounds in traditional healing practices has motivated researchers to explore their potential in clinical treatments, with notable examples such as curcumin for the treatment of AD. Despite their potential, phytochemical compounds have encountered significant challenges in neurological therapeutics. Existing data reveal that these compounds face critical obstacles similar to other active ingredients, particularly the fundamental challenge of brain penetration. This limitation has historically prevented many promising compounds from becoming effective treatments for neurodegenerative disorders. Nanoparticle (NP) technology emerges as a potential breakthrough in addressing these longstanding challenges. By providing an advanced drug delivery method, NPs offer an innovative approach to transporting therapeutic compounds across the BBB, potentially overcoming the penetration limitations that have hindered previous treatment strategies. Surprisingly, phytochemical NPs remain a relatively unexplored research domain. This review paper examines recent advancements in neurodegenerative disorder treatments, focusing on both small and large drug molecules. The manuscript particularly emphasizes the potential advantages of formulating phytochemical compounds as NP preparations. The review concludes by presenting the most recent developments in phytochemical NP formulations, highlighting their promising potential for treating neurodegenerative disorders. By exploring innovative delivery mechanisms, this paper aims to illuminate a path forward in addressing some of the most challenging neurological conditions.

## 2 Current Treatments for Noncommunicable Neurological Disorders

A wide range of treatment options for neurological disorders is available. Small drug molecules, antibodies, and stem cells have been employed for the treatment of ad [25]. However, there is no effective treatment for ad, and as a result, it is necessary to develop an effective treatment for ad. The acetylcholinesterase inhibitors (AChEIs) are cornerstone medicines that delay cognitive decline in ad. The cholinergic hypothesis is based on the progressive loss of cholinergic innervation in ad, which is important for brain functions such as memory, learning, and attention. These medicines include donepezil, galantamine, and rivastigmine. Clinical investigations in patients with ad demonstrated the benefits of donepezil and galantamine for an increase from baselines for ADAS‐cog and Mini‐Mental State Examination (MMSE) scores [26]. Aducanumab is an antibody developed by Biogen that selectively targets aggregated Aβ. Following the promising outcome of aducanumab from in vivo [27] and Phase I clinical trial [28], Biogen conducted two Phase 3 clinical trials (EMERGE and ENGAGE) to evaluate aducanumab in early ad [29]. Aducanumab was administered at a low dose of 3 mg/kg to ApoE ε4+ patients or 6 mg/kg to ApoE ε4− via intravenous infusion following dilution into saline every 4 weeks over 76 weeks (20 doses total), as well as a high dose of aducanumab at 10 mg/kg. Both dose regimens of aducanumab significantly reduced the brain amyloid load compared to placebo by week 78. Aducanumab resulted in a 22% statistically significant reduction in the decline of CDR‐SB, an 18% reduction in the decline of MMSE, and a 27% reduction in the decline in the Alzheimer's Disease Cooperative Study–Activities of Daily Living scale (ADCS‐ADL)‐MCI for the 10‐mg/kg dose regimen in the EMERGE cohort. The incidences of amyloid‐related imaging abnormalities–oedema (ARIA‐E) were much higher in both doses in both cohorts [29]. As the EMERGE trial met its primary outcome and the ENGAGE trial did not, Biogen submitted the data to the US Food and Drug Administration (FDA) for review and possible marketing approval. The FDA approved aducanumab (Aduhelm) in 2021 with a high starting price of $56,000 per year, but the price was later reduced to $28,200 [30].

Glatiramer acetate and interferons are the first approved disease‐modifying therapies for MS. [31] Disease‐modifying therapies modify the course of MS through the suppression or modulation of immune function. To achieve more effective therapies, strategies were developed to inhibit lymphocyte access to the CNS by using drugs such as natalizumab (α4 integrin antagonist) [32] and siponimod (functional antagonist of sphingosine‐1‐phosphate receptor) [33]. Autologous hematopoietic stem cell transplantation is another approach that is under investigation to induce a prolonged remission in MS patients [34].

Small molecules such as levodopa have been used for the treatment of PD since the 1960s [35]. In addition, the presence of abnormal α‐synuclein aggregates is the pathological hallmark of PD. To remove these, prasinezumab has been developed, which is a humanized monoclonal antibody targeting the C‐terminus of aggregated α‐synuclein [36]. Although the Phase II clinical trial did not find prasinezumab therapy to have a meaningful effect on global or imaging measures of PD progression compared with placebo [37], deep brain stimulation has been a useful treatment for PD when levodopa and other dopamine replacement therapies become ineffective [38]. Deep brain stimulation involves a surgical procedure to implant electrodes in the brain to stimulate subcortical structures, including the internal globus pallidus and subthalamic nucleus [39].

TBI is defined as a sudden injury that causes damage to the brain, with 69 million individuals suffering every year worldwide. Because of the mechanical damage, the breakdown of the BBB happens, as well as hemorrhage. This is known as the primary damage. However, there is secondary damage, accompanied by inflammation, ischemia, and oedema. These injuries occur within minutes of the injury, but the damage continues, which leads to the development of neuropsychiatric comorbidities. Tranexamic acid is a small molecule that is used in mild to moderate TBI to reduce the risk of death, which is administered within 3 h of the injury [40]. Additionally, mannitol and hypertonic saline are intravenously administered to reduce intracranial pressure [41]. Due to possible posttraumatic seizures, antiepileptic drugs may be administered. There are other medicines administered for the treatment of post‐TBI neuropsychiatric changes such as selective serotonin reuptake inhibitors (sertraline and citalopram), serotonin and norepinephrine re‐uptake inhibitors (milnacipran), serotonin 1A receptor partial agonist (buspirone), antipsychotics (methotrimeprazine, droperidol, haloperidol), and prazosin (to reduce the severity and frequency of nightmares associated with posttraumatic stress disorder) [42].

Stroke is defined as a focal neurological deficit that only cerebrovascular disease can explain. Ischemic stroke accounts for 60%–70% of all strokes, and it is the result of acute arterial occlusion. Intracerebral hemorrhages are caused by a vessel rupture in the brain [43]. Restoration of the cerebral perfusion is the main aim of ischemic stroke treatment. This is achieved by the administration of intravenous thrombolysis and/or endovascular thrombectomy. Intravenous administration of alteplase is approved by all regulatory agencies and is currently the only thrombolytic agent for the treatment of ischemic stroke. Tenecteplase may be used off‐label for the treatment of acute ischemic stroke [44]. Intra‐arterial therapy can be divided into chemical dissolution of clots with locally delivered thrombolytic agents or removal of the clot with a mechanical device. Intra‐arterial treatment (intra‐arterial thrombosis and/or mechanical treatment provided better outcomes than a control group that received usual care alone [45].

CNS tumors result from irregular cellular growth in the brain and spinal cord, which is associated with neurological symptoms. The age‐standardized rate of CNS cancers was 12.5 per 100,000 in 2021. [11] The treatment depends on the type of tumor, which may need a combination of surgery, chemotherapy, and radiotherapy. Patients with advanced breast cancer may develop brain metastases. Whole‐brain radiation therapy (WBRT) or surgical resection form part of the treatment [46]. Lapatinib is a tyrosine kinase inhibitor with a molecular weight of 581 g/mol and can access the normal brain and brain metastases [47]. A systemic review and meta‐analysis revealed that lapatinib yielded better survival for HER‐2+ breast cancer patients with brain metastases [48]. Temozolomide is a small‐molecule chemotherapeutic drug used for brain tumors such as glioma. A recent study found that adjuvant use of temozolomide with radiotherapy showed better survival (median overall survival 116·6 months) of patients with anaplastic glioma compared to concurrent temozolomide chemotherapy [49]. There are other medicines used for the treatment of brain metastases reviewed recently [46, 50].

**FIGURE 1:**
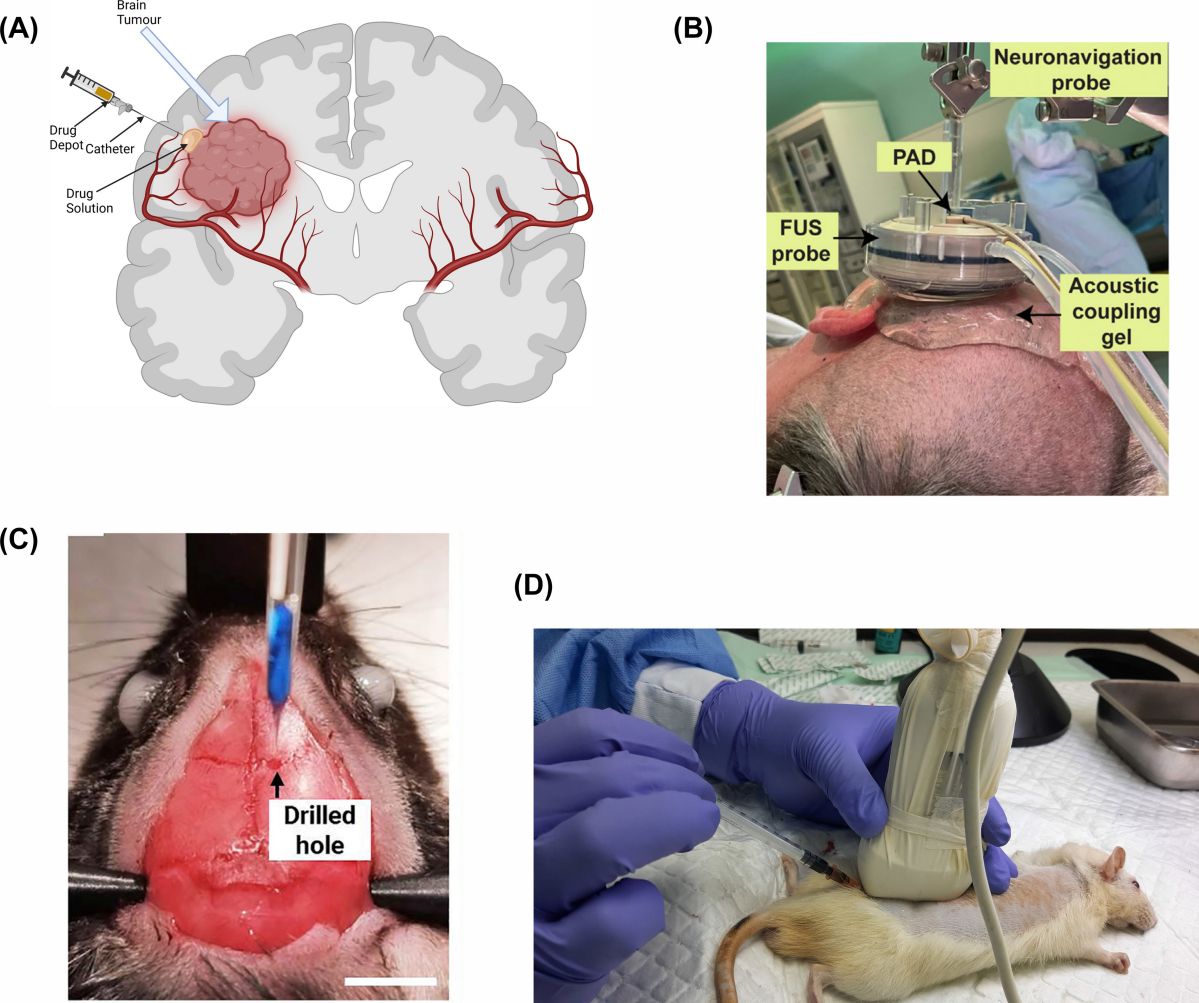
Invasive drug delivery to the brain. (A) Schematic presentation of convection‐enhanced drug delivery, (B) ultrasound‐mediated BBB disruption [53], (C) transcranial injections [55], and (D) intrathecal administration [57]. Reproduced with permission from the cited references.

In the search for more effective treatments, several techniques have been developed for both invasive and noninvasive drug delivery systems. Invasive drug delivery systems include convection‐enhanced drug delivery (Figure 1A) [51], ultrasound‐mediated BBB disruption (Figure 1B) [52, 53], transcranial injections [54], also known as stereotaxic injections [55] (Figure 1C), and intrathecal administration (Figure 1D) [56, 57]. Intrathecal administration is used for the management of therapy‐resistant pain, spasticity, and dystonia when oral therapy has not been successful [58]. Ultrasound‐mediated BBB disruption was employed for delivering antibodies to the brain [52]. The noninvasive technique is the formulation of novel and complex NPs [59] as well as novel shuttle peptides [60]. Although invasive delivery systems have been more frequently used in clinics, NP‐based formulations are making their way through clinical trials [61]. Brain mapping is done during surgery (https://www.youtube.com/watch?v=u50HPRe3rOY).

### 2.1 The Limitations of Current Treatments

Although there are several treatments for neurological disorders, the worldwide burden of neurological disorders is increasing. The medicines invented for CNS diseases have shown limited clinical efficiency. As a result, high drug doses and continuous consumption have been considered, leading to associated side effects. Aging is another factor that makes treatment challenging due to aging‐related comorbidities. For certain treatments, such as antibodies, the BBB has presented a prominent obstacle for reaching large drug molecules in the brain [52]. Intrathecal injections of methotrexate have led to death due to brain damage in leukemic patients [62]. In addition, drugs such as tranexamic acid do not reduce death in severe TBI patients with extensive intracranial hemorrhage. [63] As a result, the World Health Organization has set a new Intersectoral Global Action for improving the treatment of neurological disorders [64]. One of the objectives of this action plan is “Provide effective, timely and responsive diagnosis, treatment and care for neurological disorders” [65]. The development of targeted interventions is pivotal to promoting, improving, and monitoring brain health across the whole life [66]. The development of new treatments forms part of the targeted interventions, as well as research drug development and the development of new therapies. This includes epidemiological studies and access of all patients to new therapies [67].

The invasive drug delivery methods suffer from risks such as CSF leak (intrathecal administration) or developing meningitis [58]. The brain damage might be due to the direct contact of a medicine with neurons at high doses. The focus on ultrasound‐mediated BBB disruption requires complex considerations for each patient [68]. These include a well‐defined ultrasound acoustic dose to reduce variability in BBB disruption. Acoustic waves could reflect from different surfaces and interfere with other waves. Furthermore, the BBB has the physiological role of protecting the brain from harmful compounds in the brain. A small area of hypo‐intensities has been reported with focused‐ultrasound BBB opening, which disappeared after 24 h [69]. These could be microhemorrhages around the BBB [69]. Therefore, long‐term safety should be evaluated for opening the CNS barriers. In addition, during the focused ultrasound BBB opening, the presence of an anesthesiologist may be required [52], or the patient is under anesthesia [53]. For transcranial drug delivery injections, precise brain mapping and delivery to the site of the lesion are required, which limits the application of this method [70]. It should be added that a surgical procedure is required for transcranial administration, which would limit the number of drug administrations. Nonminimally/minimally invasive techniques include intravenous injections of NPs, exosomes, shuttle peptides, and stem cells.

The unclear approval process of the FDA and the drug's price prompted criticism and led Congress and the FDA to launch investigations into relations between Biogen and key figures at the regulatory agency [71]. The investigations found that the FDA worked unusually closely with Biogen staff in a “collaborative workstream” that massaged the poor trial results and changed study endpoints. Unexpectedly, the FDA considered only the reduction of brain amyloid load as the evidence of clinical benefit, which was never accepted by the FDA statisticians as well as nonsignificant clinical benefits. As a result, they were excluded from meetings. In addition, a group of international researchers, clinicians, and policy experts met on December 15, 2021, and they voted unanimously to recommend that the FDA withdraw its approval for aducanumab [72]. Following these investigations, Biogen decided to withdraw aducanumab from the European market on April 20, 2022. Furthermore, Biogen announced in January 2024 that Aduhelm would be discontinued in November 2024.

## 3 NPs for the Treatment of Brain Disease

Nanotechnology has gained significant attention for the treatment of neurological disorders. NPs are materials with overall dimensions in the range of 10 to 1000 nm [73]. NPs can have different shapes, such as spheres [74], rods [75], fibers [76], or irregular [77]. The NPs can have filled or core–shell structures [78]. Several types of NPs have been developed for drug delivery to the brain, such as polymeric NPs [79], liposomes [80], exosomes [81], and peptide‐based NPs [82]. There are several advantages of using NPs for drug delivery to the brain, as briefly explained below.

### 3.1 Facilitating Crossing the Blood–Brain Barrier

**FIGURE 2:**
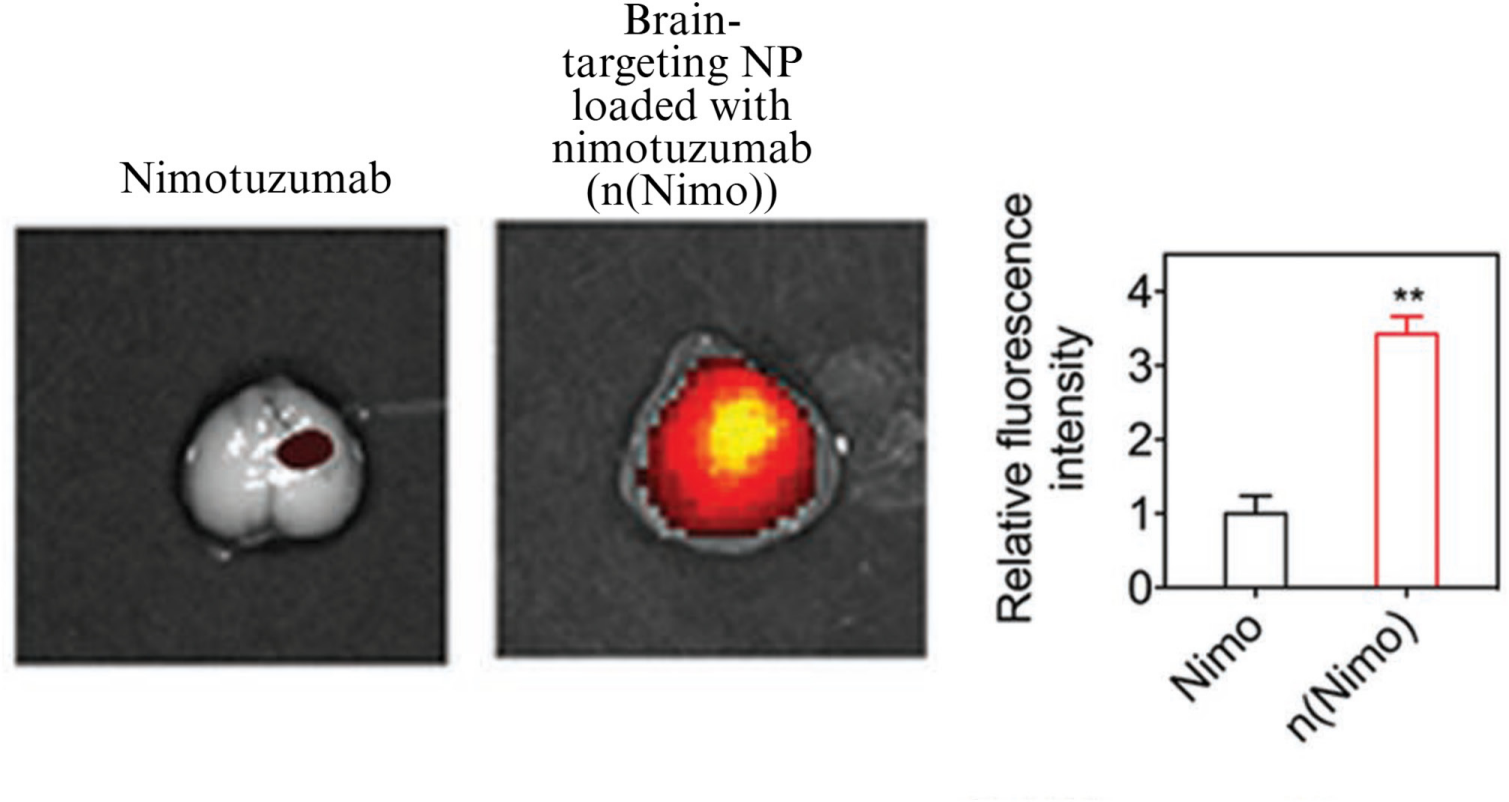
Representative ex vivo fluorescence images of glioma‐bearing brain tissues 10 days after one injection (5 mg kg^−1^) of nimotuzumab or brain‐targeting NPs loaded with nimotuzumab, n(Nimo), labeled with Cy5.5. The histogram compares the relative fluorescence intensity of the tumor‐bearing brain tissue. **p < 0.01 (two‐tailed Student's *t*‐test). Data represent mean ± standard error of the mean (*n* = 3). Reproduced with permission from [83].

NPs can encapsulate large hydrophilic molecules such as small interfering RNA (siRNA) or monoclonal antibodies and cross the BBB and deliver to the brain (Figure 2) following a systemic administration [83]. Furthermore, NPs can protect sensitive compounds such as siRNA from enzymes in the blood by providing a core–shell structure that the active ingredient is encapsulated within the NP [84, 85]. NPs can be used as a carrier to deliver small hydrophilic molecules such as curcumin to the brain [86, 87]. NPs can be functionalized with ligands that bind to specific receptors on brain cells. This targeting ability allows for more precise drug delivery, potentially increasing therapeutic efficacy while reducing off‐target effects. For instance, fluorinated polyethylenimine NPs have been designed to target microglia in the brain following intravenous administration and deliver TREM2‐encoding plasmid for treating neuroinflammation in neurodegenerative diseases such as AD [88].

### 3.2 Sustained Release of Drug

**FIGURE 3:**
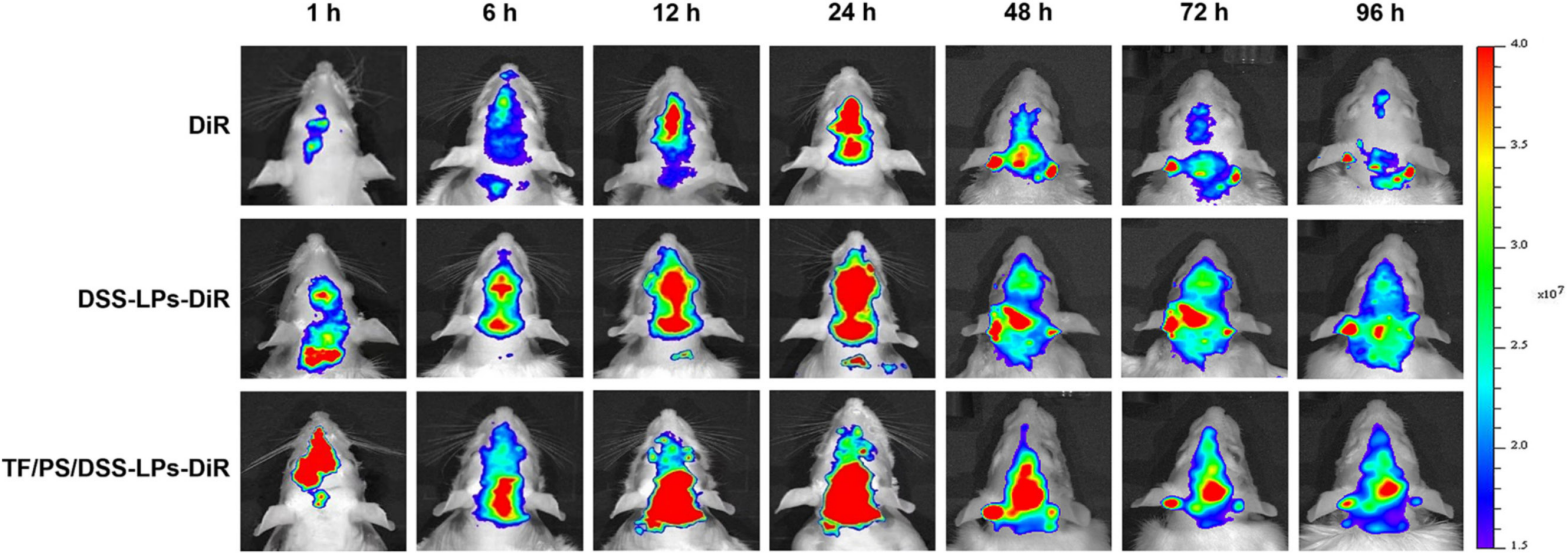
Fluorescence analysis of targeting rat brains following iv injection of DiR, liposomes (LPs) loaded with danshensu (DSS) and DiR (DSS‐LPs‐DiR), and liposomes decorated with transferrin (TF) and phosphatidylserine (PS) and loaded with DSS and DiR (TF/PS/DSS/‐LPs‐DiR). Reproduced with permission from [89].

Furthermore, NPs can be engineered to release drugs at a specific rate over an extended period in the brain. This controlled release can maintain therapeutic drug concentrations in the brain for longer durations, potentially improving treatment efficacy and reducing the need for frequent dosing. For example, dual‐targeting liposomes were formulated by encapsulating danshensu. These liposomes are surface‐decorated with transferrin molecules to cross the BBB and phosphatidylserine to target microglia. The liposomes released danshensu gradually over 96 h in vitro and in vivo (Figure 3) [89].

### 3.3 Protection of Sensitive Drugs

siRNAs have the potential for therapeutic application in brain diseases, such as glioblastoma [90], ad [91], PD [92], TBI [93], and stroke [94]. As siRNAs are subject to rapid enzymatic degradation in serum endonucleases or exonucleases [95], NPs can encapsulate siRNAs and protect them from enzymatic degradation. For example, Raja et al. 2015 encapsulated siRNA within chitosan NPs. To evaluate the protection of siRNA, siRNA‐loaded chitosan NPs were exposed to RPMI cell culture media with 10% FBS. It was found that most of the naked siRNA was degraded within 48 h of exposure to FBS, whereas encapsulated siRNA exhibited more stability [96]. Proteins are another class of biologics that may require protection against proteases within the physiological environment. For example, the enhanced green fluorescence protein (EGFP) is used for imaging techniques with the advantage of low toxicity. However, EGFP can be degraded by proteases. It has been shown that encapsulation of EGFP within silica NPs improved the stability of EGFP against proteases [97]. Insulin is another protein that is unstable in an acidic environment. Encapsulation of insulin with polymeric NPs allowed protection of insulin from the gastric enzymes and pH and provided an opportunity to significantly lower blood glucose levels in diabetic rats compared to oral unprotected insulin solution [98].

## 4 Reduced Side Effects

NPs enable more targeted delivery and potentially lower the overall dose required, which can help reduce systemic side effects. This is particularly important for drugs with narrow therapeutic windows or significant toxicity profiles, such as Doxil, which is a liposomal formulation of doxorubicin [99]. This is because small drug molecules may not have selectivity for the particular target tissue and remain within the systemic circulation with back‐and‐forth diffusion into several off‐target organs. Although NPs reduce widespread systemic distribution of the drug molecules, this targeted delivery allowed achieving high maximum tolerated dose for paclitaxel by using a Cremophor‐free, protein‐stabilized, NP formulation of paclitaxel [100]. Therefore, NP‐based delivery systems can also help minimize systemic side effects often associated with brain‐targeted therapies such as glioblastoma multiform [101]. It should be noted that NPs themselves may impose toxicity [102, 103]. Carbon nanotubes have attracted a great deal of research and interest as NP‐based drug delivery. For example, carbon nanotubes can cross the BBB without the need for a brain‐targeting ligand [104]. Carbon nanotubes were employed as carriers for the delivery of acetylcholine into the brain for treating animal models [105]. However, there are reports of toxic effects following exposure to carbon nanotubes [102].

## 5 Improved Solubility and Stability

Combinatorial chemistry has been employed for drug development, both for lead discovery and optimization [106]. In this approach, a diverse range of compounds is synthesized by applying techniques such as Houghten's tea bag technology to form a chemical library [107]. Then the compounds are screened against a variety of biological targets such as cytotoxicity, cell signaling, and binding to the target protein. Over 40% of drug molecules that are identified through this combinatorial screening program are poorly water soluble [108]. This causes difficulties in formulating the active ingredient using conventional techniques, as drug bioavailability may not be sufficient to achieve therapeutic targets. The formulation of nanocrystals has been employed to overcome the poor solubility of active ingredients. The surface area of solid particles significantly increases by reducing particle size, which enhances the dissolution rate. In addition, the formation of nanocrystals improves saturation solubility through changes in the physicochemical properties of the compound such as crystalline structure [109]. Formation of fenofibrate nanocrystals improved its solubility from 0.3 to 5.7 μg/mL [110].

## 6 Potential for Noninvasive Administration

The olfactory of the nasal cavity contains nerves that provide direct access to the CNS by bypassing the BBB. Lipophilic and small drug molecules may get access to the CNS through paracellular diffusion along the olfactory sensory neurons as well as crossing the intracellular pathways of the neuron cells. However, drug molecules with masses over 1000 Da have low permeability through the sensory neurons, and therefore, the absorption is reduced. NP formulations have allowed the delivery of large drug molecules to the brain [111] such as glatiramer acetate for the treatment of MS in animal models. Glatiramer acetate (a random polypeptide) was encapsulated in lipid NPs and administered nasally to experimental autoimmune encephalomyelitis (EAE) mice. The clinical scores of EAE mice were improved significantly compared to control EAE mice that had only glatiramer administration. In addition, curcumin (a poorly water‐soluble drug) was encapsulated in lactoferrin and administered intranasally to wild‐type rats [112]. Considerable amounts of the NPs were detected in the brains following intranasal administration. This nasal delivery provides a noninvasive delivery route to the brain, which may be considered a more patient‐friendly and safer alternative to invasive methods such as intravenous or intracranial injections.

Oral delivery of insulin has been widely investigated, with recent positive results from insulin oral capsules [113]. A Phase 2 clinical trial showed improvements in Type 2 diabetic patients using oral insulin 338 (I338). This is a long‐acting, basal insulin analogue formulated in a tablet with the absorption enhancer sodium caprate [114]. Although the clinical trial met the primary outcomes, further development of this particular oral insulin project was discontinued. This was because I338 doses were high and, therefore, production of the required quantities of I338 for wide public use was considered not commercially viable [114]. NP formulation of insulin may provide an oral administration route with reduced amounts of insulin. Insulin NP formulations for oral delivery have been reviewed recently [115].

## 7 Multifunctional Capabilities

NPs can be designed to serve multiple functions simultaneously. For example, they can carry both therapeutic agents and imaging contrast agents, allowing for real‐time monitoring of drug delivery and treatment response. These formulations are known as theranostic (theragnostic) NPs [116]. Lam et al. 2018 formulated pegylated liposomes, which were functionalized with transferrin (BBB targeting component). The lipid structure of the liposomes also contained Cy5.5‐labeled lipids, which allowed tracing the liposomes in the brain of animal models using an IVIS instrument. These liposomes were loaded with two drugs: temozolomide and a bromodomain inhibitor. The theranostic liposomes confirmed targeting glioblastoma and prolonged the animal's survival [117]. Carbon nanotubes provide a platform for developing brain‐targeting theranostic NPs. Costal et al. 2018 employed multiwalled carbon nanotubes (MWNTs), which were functionalized with derivatives of Pittsburgh Compound B (PiB) not only to target Aβ plaques in AD but also to allow visualization of targeting the brain by SPECT/CT imaging technique [118]. These NPs took advantage of the intrinsic property of carbon nanotubes crossing the BBB. The SPECT/CT confirmed the accumulation of functionalized MWNTs in the brain. The NPs achieved up to 1% of the injected dose accumulation per gram of the brain. Gold NPs also provide a platform for the formulation of multifunctional formulations [119]. For example, Mirrahimi et al. 2019 developed gold NPs that carried cisplatin, and these were employed for the treatment of the CT26 colorectal tumor model. The diseased animals were treated with 532 nm laser irradiation and received dramatically higher thermal doses due to the optical absorption properties of AuNPs. The AuNPs provided a combined action of chemo‐photothermal therapy, and the tumor growth was significantly less than control animals [120].

## 8 Phytochemical NPs Hold Promise

Phytochemical compounds have been considered for the treatment of brain diseases. For example, curcumin is a polyphenol compound obtained from *Curcuma longa* with a wide range of applications from food to the textile industry. Curcumin has been demonstrated to reduce Aβ burden in the brain of aged Tg2576 mice by inhibiting Aβ aggregation and fibril formation [121]. Curcumin was generally well tolerated in a Phase I clinical trial for the treatment of mild‐to‐moderate probable Alzheimer's disease at a dose of 2 g/day [122]. However, the Alzheimer's Disease Assessment Scale—Cognitive Subscale (adAS‐Cog) was significantly different from the placebo group at 24 weeks. A similar trend was observed for the MMSE score. Furthermore, the plasma and CSF levels of curcumin were determined using liquid chromatography/tandem mass spectrometry (LC/MS/MS). Curcumin and its metabolites were detected in the plasma of the treatment group, but not in the CSF. Therefore, the lack of efficacy from curcumin capsules could be due to the poor penetration of curcumin into the CNS [123] and rapid clearance from the body with a half‐life of 6–7 h [124].

Quercetin is a flavonoid found in fruits and vegetables such as onions and apples. Quercetin was dissolved in phosphate buffer saline containing 0.1% dimethyl sulfoxide and intraperitoneally administered to 3xTg‐ad mice at a dose of 25 mg/kg every 48 h for three consecutive months [125]. Quercetin significantly reduced the Aβ load in the CA1, the subiculum, the entorhinal cortex, and the amygdala regions of the transgenic mouse brains. Also, quercetin decreased microgliosis in the hippocampus region of 3xTg‐ad mice. Quercetin significantly reduced microglia activation compared to the vehicle‐treated transgenic mice. Furthermore, quercetin significantly improved the memory function of the transgenic mice compared to the vehicle‐treated control group. Quercetin plus dasatinib are evaluated in a clinical trial with the title of Senolytic Therapy to Modulate the Progression of Alzheimer's Disease (SToMP‐ad) Study (SToMP‐ad) and trial identifier of NCT04685590. The clinical trial is currently recruiting. Dasatinib is given as one 100‐mg capsule daily for two consecutive days, and quercetin will be given as four 250‐mg capsules daily (total 1000 mg daily) for the same two consecutive days. Both are administered orally. This treatment is based on the removal of senescent cells using dasatinib and quercetin from the CNS [126]. Early outcomes were released recently [126]. The CNS penetrations of dasatinib and quercetin were assessed by evaluating drug levels in the CSF of the participants using high‐performance liquid chromatography with tandem mass spectrometry. Dasatinib levels were detected in the CSF of four participants, ranging from 0.281 to 0.536 ng/mL, but quercetin was not detected in five participants who completed the trial. Cognitive and neuroimaging endpoints did not significantly differ from the baseline to posttreatment after 12 weeks of treatment [126].

Huperzine A is an alkaloid isolated from the Chinese folk medicine *Huperzia serrata* . It is a reversible and selective inhibitor of AChE and has been used in the clinical treatment of AD in China [127]. Oral administration of huperzine A significantly reduced escape latency in the mouse model of cerebral ischemia. Huperzine was administered at the dose of 0.2 mg/kg, once per day, starting 2 days before surgery, and lasting for 7 days after surgery [128]. In addition, bryostatin is a natural product extracted from the bryozoan *Bugula neritina* [129] and has shown positive outcomes in clinical trials for the treatment of AD [130, 131].

Generally, natural products did not show desired significant therapeutic improvements for the treatment of brain disorders in clinical trials [132]. Therefore, several NP formulations were developed, which showed much improved therapeutic efficacy in vivo compared to the base natural product, although not many of them are in clinical trials. So far, only the APH‐1105 NP formulation has been studied in clinical trials, which is administered intranasally. APH‐1105 is a nanoformulation of a potent analogue of Bryostatin 1 [133]. This is a modulator of the α‐secretase [134], which is an enzyme that cleaves the amyloid precursor protein into a more soluble compound, allowing faster clearance from the brain, and does not lead to the formation of insoluble amyloid plaques [135]. Detailed examples are given in the following for NP formulations containing natural products.

Lipid NPs were developed that encapsulated quercetin for the treatment of AD. The NPs were functionalized with transferrin to facilitate crossing the BBB [136]. In vitro studies demonstrated the capacity of the NPs to inhibit fibril formation. Similarly, lipid NPs were developed that were functionalized with RVG29 peptide. The NPs were loaded with quercetin. These NPs also inhibited the formation of Aβ fibrils in vitro [137]. Quercetin nanocrystals were developed by an evaporation precipitation of nanosuspension method [138]. The nanocrystal formulation was orally administered at a dose of 10 or 25 mg/kg to 6‐hydroxydopamine (6‐OHDA)–induced Parkinson‐like rat models. Stereotaxic injection of 6‐OHDA‐induced lesioned rats showed a significant increase in rotations compared to a control group. Administration of quercetin nanocrystals at a 25‐mg/kg dose significantly reduced the number of rotations compared to untreated animals (i.e., with the brain lesions).

Copolymers of poly (ethylene oxide)‐*b*‐poly(*ε*‐caprolactone) (PEO‐*b*‐PCL) self‐assembled to NPs loading curcumin and L‐DOPA via nanoprecipitation and solvent displacement method [139]. The NPs were coated with glutathione to facilitate crossing the NPs through the BBB. These NPs were formulated for the treatment of PD. The in vitro studies demonstrated the biocompatibility of the NPs toward Vero and PC12 cells. Polydopamine‐based curcumin‐loaded NPs (RPC NPs) were decorated with a peptide obtained from rabies virus glycoprotein (RVG) 29 to target the brain following intravenous administration for treating 6‐OHDA‐PD animal models [140]. PC‐NPs reached the brain in a time‐dependent manner, with 12 h postadministration reaching the maximum. RPC NPs significantly reduced dopaminergic neuron damage and improved the neurobehavioral abnormalities (measured by rotarod, pole, swimming, and open‐field tests) in PD mice.

**FIGURE 4:**
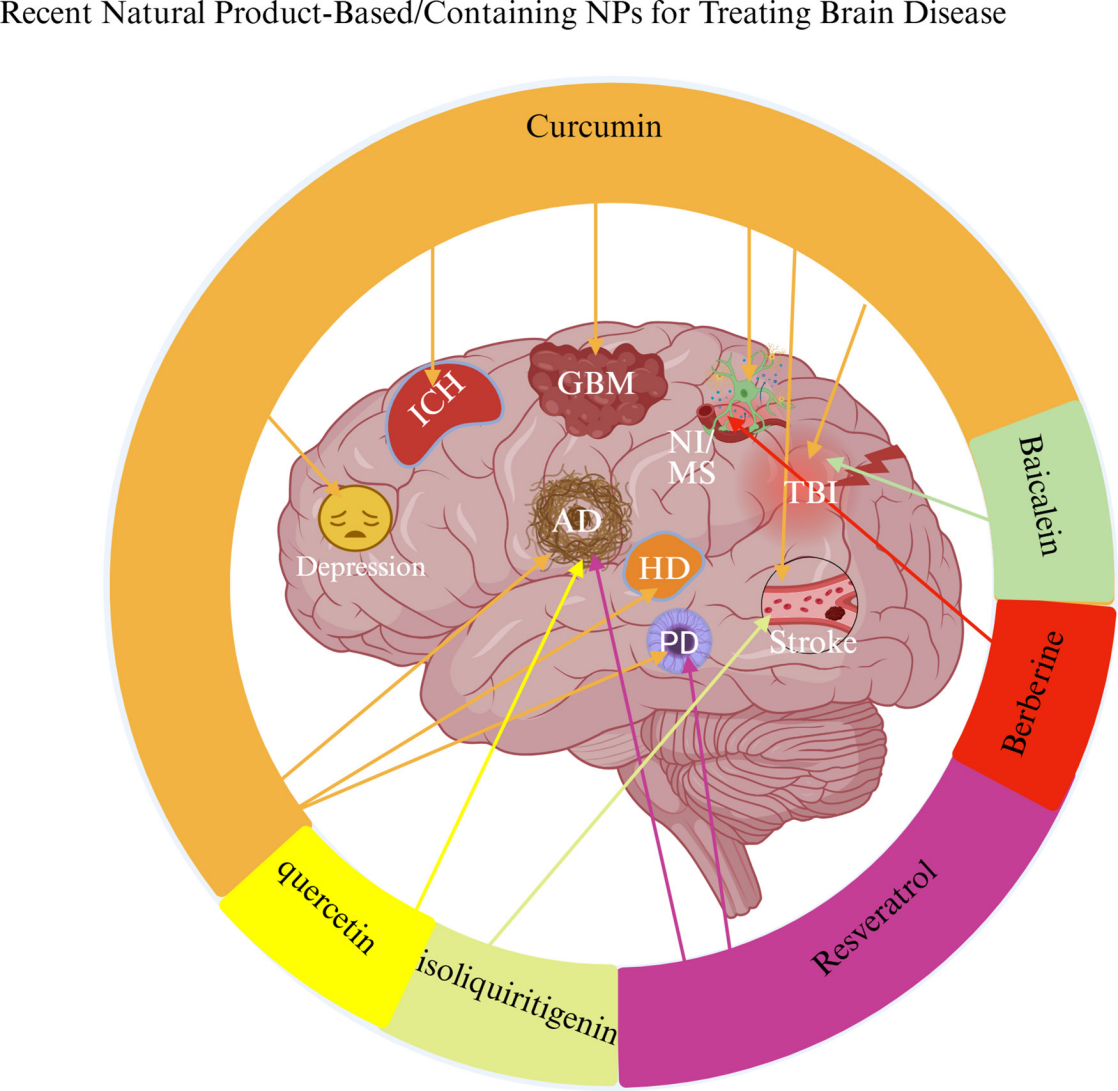
Recent natural product–based nanoparticle formulations developed for the treatment of brain diseases.

Figure 4 schematically summarizes recent applications of natural product–based NP formulations for the treatment of brain diseases, and Table 1 provides further details about these recent investigations.

**TABLE 1:** Summaries of NP formulations encapsulating natural products for the treatment of neurodegenerative diseases.

Neurol. dis.	NP material	Active ingredient	NP size/charge	BBB crossing strategy/main findings	Ref.
AD	PLGA 50:50	Curcumin	101 nm/54 mv	In vitro tests on SK‐N‐SH cell line, ↓*GLX* and *TRX* gene expressions	[141]
	^a^Ce/Zr‐MOF	Curcumin	126 nm/43 mV	Lactoferrin/↓Aβ_1–42_ in the brain of ad mice.	[142]
	NLC	Resveratrol	160 nm/25 mV	RVG29 peptide used/↑cognitive ability, ↓Aβ_42_ levels in the brain of ad (APP/PS1) mice.	[143]
	SLN	Resveratrol	104 nm/−3 mV	No BBB crossing strategy/↑GSH levels in the brain, ↓escape latency in ad rat models.	[144]
	Curcumin NPs	Curcumin	ND	No BBB crossing strategy/↑AchE activity, ↓TNF‐α levels in the hippocampus of the ad rat model	[145]
	Hollow meso‐porous Prussian blue coated with RBC membrane	Curcumin + miRNA‐124	147 nm/−11 mv	CKLVFFAED peptide used/↑memory function, ↓proinflammatory secretion from Aβ‐activated microglia	[146]
	PEG‐pLys polymer	Curcumin	65 nm/ND	KLVFFAED peptide used to cross the BBB and targeting Aβ plaques/↑memory function, ↓Aβ burden in APP/PS1 mice	[147]
	Chitosan	Resveratrol	20 nm/35 mv	TG peptide used/↑GLUT1 expression in the ad mice, ↓IL‐1β and TNFα levels in the brain of obesity related ad mice	[148]
	Manganese‐doped cerium dioxide	Resveratrol	120 nm/−44 mV	No BBB crossing strategy/↑GSH‐PX activity in the brain of ad mice/↓Aβ load in the brain of ad mouse	[149]
	NLC	Curcumin	205 nm/ND	No BBB crossing strategy/↑brain curcumin concentration compared to control	[87]
Experimental dementia	Pluronic micelles	Resveratrol	33 nm/−4 mV	No BBB crossing strategy/↑cAMP‐response element‐binding protein expression in the cortex and the hippocampus of rats, ↓AchE content in the hippocampus of the rat models	[150]
PD	PS‐PCL	Resveratrol + ceftriaxone	200 nm/−45 mV	Leptin + transferrin/↑Bcl‐2 and ↓α‐syn fibril formation in the substantia nigra of PD rat models	[151]
	LN (GMO)	Curcumin + piperine	93 nm/−31 mV	No BBB crossing strategy/↑PC12 cell survival, ↓functional deficits in a rotenone‐induced PD mouse model	[152]
Stroke	Gelatin	Curcumin	141 nm/ND	RVG29 peptide used/↑behavioral functions, ↓IBA‐1 expressing microglia in the MACO rat model	[153]
	PLGA	Curcumin	220 nm/−20 mV	No BBB crossing strategy/↑neurological scores, ↓mRNA TNF‐α in the brain of the SAH rat model	[154]
	Lipid	Curcumin	134 nm/ND	No BBB crossing strategy/↑memory consolidation in MWM test in rats with (I/R) injury	[155]
	DSPE‐PEG_2000_	Isoliquiritigenin	41 nm/−34 mV	Angiopep‐2 peptide used/↑blood circulation time, ↓expression of LC3 protein	[156]
Heat shock	PAMAM	Curcumin	90 nm/7 mV	No BBB crossing strategy/↑exploration activity (e.g., recognition index), ↓TNF‐α, and IL‐1β in the brain of the heat shock mouse model	[143]
Antidepressant	SLN	Curcumin+HU‐211	59 nm/−22 mV	No BBB crossing strategy/↑dopamine levels in PC 12 cells, ↓the duration of immobility in the major depression mouse model	[157]
HD	SLN	Curcumin	148 nm/ND	No BBB crossing strategy/↑brain SDH activity compared to control animal model, ↓reduced the number of paw slips compared to control animal models, allowing walking through narrow beams	[158]
IH	SLN	Curcumin + TGF‐β1 siRNA	125 nm/39 mV	IN delivery/↑intracellular antioxidant, ↓IL‐1β, IL‐6, and TNF‐α in the brain of the animal model	[159]
TBI	MnO_2_–Au–mSiO_2_ nanomotors	Curcumin	100 nm/−10 mV	No BBB crossing strategy/↑M2/M1 microglia phenotype ratio in BV2 cell line, ↓escape latency in the animal model	[160]
	Poly (propylene sulfide)_120_	Curcumin	100 nm/−13 mV	CAQK peptide used/↑brain GSH‐px levels, ↑brain IL‐1β, and TNF‐α levels	[161]
	As supplement	Curcumin	ND	500 mg orally/8 h/in TBI patients/↓brain oedema	[132]
	Liposomes	Baicalein	158 nm/−28 mV	No BBB crossing strategy/↑BBB integrity/↓brain IL‐1β in TBI mice	[162]
GBM	cRGD‐PEG‐PCL	Curcumin + DOX	76 nm/0 mV	cRGD peptide used/↑cell apoptosis in GL261 cells, ↓tumor volume growth	[163]
NI	Liposomes (DSPE‐PEG_2000_‐ANG)	Resveratrol	85 nm/−2 mV	Angiopep‐2 peptide used/↑NeuN marker (neuronal density in the hippocampus)/↓IL‐1β and TNF‐α levels in the brain of aged mice	[164]
MS	Dendrosomal curcumin	Curcumin	142 nm/−7 mV [165]	No BBB crossing strategy/↑mean clinical score in EAE rats/↓IL‐1β and TNF‐α levels in the spinal cord of EAE rats	[166]
	Iron‐oxide	Berberine	22 nm/ND	No BBB crossing strategy/↑spatial memory in EAE rats/↓serum INF‐γ levels	[167]

Abbreviations: ad: Alzheimer's disease, GBM: glioblastoma, HD: Huntington's disease, IH: intracerebral hemorrhage, MS: multiple sclerosis, NI: neuroinflammation, PD: Parkinson's disease.

^a^ Ce/Zr‐MOF is a type of bimetallic mixed metal–organic framework.

## 9 Critical Evaluation of the Challenges in Translating

The translation of phytochemical NPs from laboratory bench to clinical bedside represents one of the most promising yet challenging frontiers in modern nanomedicine. Although these bioactive compounds derived from plants offer tremendous therapeutic potential, their successful clinical implementation faces numerous complex obstacles and, unfortunately, may involve more than synthetic compounds, which should be systematically addressed. The primary hurdle lies in achieving consistent, reproducible manufacturing processes. Because the active ingredient is sourced from plants, these phytochemicals exhibit inherent batch‐to‐batch variability due to factors such as plant source, extraction methods, seasonal variations, and geographical differences [168]. Even the same plants may not produce the same phytochemical compounds at every harvesting time. The use of multivariate analysis has been suggested to identify the source of variation, such as packing behavior [169]. Furthermore, the harvested plant may promote degradation of the target compound and microbial contamination due to relatively high amounts of moisture [170]. When incorporated into NP formulations, factors such as variations in plant species, growth conditions, and extraction methods have been identified as contributing factors to inconsistencies in the properties and potentially the performance of NP formulations [171]. For example, ZnO NPs extracted from *Punica granatum* peel and *coffee* ground extracts did not show antibacterial activity against several bacterial strains, such as *Pseudomonas aeruginosa* , whereas chemically synthesized ZnO NPs showed inhibitory effects [172]. This variability makes it challenging to establish standardized protocols that meet regulatory requirements. Extraction may form part of the manufacturing process. It is the process of separating bioactive components of plants using selective solvents by applying standard procedures. The extracted ingredients are relatively complex mixtures of bioactive constituents. Ideally, the chosen solvent should be nontoxic [173]. As part of regulatory requirements, the impurities should be identified and quantified in NP formulations. The formulation of phytochemical NPs has the potential to impact the solubility and pharmacokinetics of phytochemicals. Reducing the size of phytochemical crystals to nanometer size (enhancing surface area) would increase the solubility of the compound and affect the absorption rate in the GI [174]. For example, the solubility of resveratrol was increased by formulating it as NPs using a thin film rehydration technique [175]. Resveratrol NPs showed improved efficacy in the EAE mice compared to resveratrol solution [175]. In terms of pharmacokinetics, paclitaxel was encapsulated in the polymeric micelles of Pluronic 123 [176]. Paclitaxel‐loaded micelles had an average size of 25 nm. Paclitaxel‐loaded micelles were administered intravenously into rats, and *t*_1/2β_ was 2.50 ± 0.63 h for Taxol injection (solution formulation) and 5.85 ± 1.52 h for the micelle formulation, indicating a 2.3‐fold increase for the micelle formulation. In addition, the AUC_0–8h_ was 1007.9 ± 192.6 μg·h/L for Taxol injection, whereas the AUC was 2916.8 ± 873.6 μg·h/L for the micelle formulation, a 2.9‐fold increase for the micelle formulation. The hydrophilic shell of the micelles avoided uptake by the reticuloendothelial system as well as rapid clearance by the kidneys [176]. In terms of translation into clinical trials, NP formulations face tougher challenges compared to classical formulations [177], in particular if the active ingredient (including phytochemicals) is not an approved drug substance. For example, for NP formulations, a well‐defined manufacturing process is needed with its associated process controls. This is to ensure that an acceptable product is produced on a consistent basis, as small changes to block copolymer micelle products may significantly influence their performance [178]. The economic landscape presents substantial challenges for clinical translation, as researchers need to choose particular plants known for their high concentrations of phytochemicals such as polyphenols or flavonoids. In addition, well‐known techniques should be employed, such as maceration and reflux, to extract the desired phytochemical ingredients [179]. Furthermore, the use of phytochemicals as a source of active ingredients may compete with food production [179]. Although these seem like challenges, phytochemicals are often derived from natural sources, making them potentially more cost‐effective than synthetic compounds [174]. Another challenge in the development of phytochemical NPs is related to poor financial support [180], perhaps due to a lack of strong intellectual property protection [181]. As part of the future perspectives, the phytochemical extracts/plant‐based NPs should be regulated through official controls such as the US Food and Drug Administration or the European Medicines Agency for rigorous manufacturing standards [180]. This is because there is a concern about the lack of official information regarding the actual toxicity of many extracts, as adverse effects may be caused by the misuse of medicinal plants, affecting public health [182]. Therefore, the successful clinical translation of phytochemical NPs demands innovative solutions that may not be common with synthetic active ingredients. This shift requires balancing the practical feasibility of these promising phytochemical NPs with whether they can fulfill their potential in providing novel solutions to human neurodegenerative diseases.

## 10 Safety Profiles and Potential Toxicity of Phytochemical NPs

As discussed above, the aim of phytochemical NPs has been to encapsulate phytochemicals in a nanoparticulate formulation. This has been to reduce the systemic toxicity of the phytochemical compounds such as paclitaxel. It is not expected that NPs encapsulating phytochemical compounds will exert different toxicity compared to synthetic active ingredients. Therefore, typical adverse events such as infusion site reactions have been reported for NPs encapsulating phytochemicals [183, 184]. NPs can exert immunotoxicity by interacting with the immune system in several ways, such as inducing the release of proinflammatory cytokines [185]. As an example, nab‐paclitaxel (NP‐bound paclitaxel) induced cytokine release syndrome in clinic [186]. However, flavonoid‐encapsulated NPs significantly downregulated proinflammatory cytokines such as TNF‐α and IL‐1β [187]. Plant‐derived NPs have achieved significant attention recently due to their potential pharmacological applications, such as antimicrobial activity [188], and plant‐derived nanovesicles have demonstrated low immunogenicity [189]. However, strawberry‐derived vesicles carried protein sequences that were homologous to known allergens [190].

## 11 Conclusion and Future Perspective

Conventional treatments often fail to adequately slow the progression of neurodegenerative diseases. The BBB and the complexity of neurodegenerative diseases have been major obstacles to achieving desired therapeutic outcomes. Techniques such as convection‐enhanced delivery and ultrasound‐mediated BBB disruption have been employed to improve the quantity of active ingredients reaching target zones in the brain. Although these represent improvements, significant therapeutic outcomes have not been achieved, and techniques such as convection‐enhanced delivery remain invasive. Furthermore, surgical removal of brain tumors or application of convection‐enhanced techniques fail to eradicate all cancerous cells. These drawbacks have prompted the development of novel NP‐based formulations, which can reach the brain via minimally invasive routes such as intravenous administration and specifically target diseased cells/neurons in the brain. Although several NP formulations are commercially available, none specifically target the brain. Most NP formulations under development aim to enhance the delivery of highly active ingredients across the BBB. Natural products are also being formulated as NP delivery systems for brain disorders. These compounds offer potential safety advantages, having been used in traditional medicine for extended periods. Multiple in vivo studies have demonstrated the efficacy and safety of natural product–based NP formulations for treating brain diseases. Future research must address regulatory requirements for NP formulations to advance these promising treatments into clinical trials. For formulation scientists, understanding regulatory frameworks and conducting appropriate compliance testing represents a critical step in this development pathway.

## Disclosure

The authors declare that this work has not been published in any other journal.

## Data Availability

Data sharing is not applicable to this article as no new data were created or analyzed in this work.
